# Artificial Intelligence is Irreversibly Bound to Academic Publishing — ChatGPT is Cleared for Scientific Writing and Peer Review

**DOI:** 10.21470/1678-9741-2023-0963

**Published:** 2023-08-31

**Authors:** Walter J. Gomes, Paulo R. B. Evora, Solange Guizilini

**Affiliations:** 1 Cardiovascular Surgery Discipline, Escola Paulista de Medicina, Universidade Federal de São Paulo, São Paulo, São Paulo, Brazil.; 2 Cardiology Postgraduate Program, Escola Paulista de Medicina, Universidade Federal de São Paulo, São Paulo, São Paulo, Brazil.; 3 Hospital das Clínicas da Faculdade de Medicina de Ribeirão Preto da Universidade de São Paulo (HCFMRP-USP), Ribeirão Preto, São Paulo, Brazil.; 4 Department of Human Motion Sciences, Universidade Federal de São Paulo, São Paulo, São Paulo, Brazil.

Artificial intelligence (AI) has recently made a nearly abrupt entrance into our routine daily life. Freely accessible, it makes up a disruptive technology steadily shaking established fundaments of our society and is thought to be here to stay.

ChatGPT, the prominent free chatbot that uses natural language processing, was released in November 2022 by OpenAI, swiftly storming the internet and prompting users to apply for presumed unlimited purposes. AI specialists consider that ChatGPT has the potential to revolutionize how users interact with chatbots and AI as a whole^[1,2]^.

Artificially intelligent computer systems are used extensively in medical sciences. Currently, the most common roles for AI in medical settings are clinical decision support and imaging analysis. Common applications for AI include disease detection and diagnosis, personalized disease treatment, accelerated drug discovery and development, telemedicine, improving patient safety with error reduction, improving communication between physician and patient, transcribing medical documents, remotely treating patients, and others^[3]^.

Not surprisingly, ChatGPT has been used by researchers in generating content for academic publishing. The AI tool has received recommendations for its use in scientific writing, as stated by the International Committee of Medical Journal Editors (ICMJE)^[4]^, and additionally for manuscript peer review, as indicated by the World Association of Medical Editors (WAME)^[5]^.

Consistent with the announcement, at submission, the authors are required by the journal to disclose whether AI-assisted technologies were used in the production of the submitted manuscript. The authors should describe how chatbots have been used and they should not be included in the authorship because they cannot be responsible for the work’s accuracy, integrity, and originality, making humans entirely accountable for the submitted material. A special mention for carefully reviewing the submitted content is stressed, given the possible incorrect, incomplete, or biased information generated by the chatbot.

The statements from the ICMJE^[4]^ and WAME^[5]^ recognizes the potential of AI language models in scientific manuscript writing. It may indicate a shift in how scientific writing is approached and the recognition of AI language models as valuable tools in the research process.

Using AI language models in scientific manuscript writing can offer several advantages. These models can assist researchers in generating high-quality drafts and offering suggestions for content organization, grammar, and style. They can help streamline the writing process by providing a starting point or helping overcome writer’s block. Additionally, AI language models can potentially improve manuscript quality by identifying inconsistencies, errors, or gaps in the content.

However, AI models may inadvertently reproduce biases or inaccuracies present in the training data. Researchers should be cautious and critically evaluate the content generated by these models to ensure scientific accuracy, consistency, and adherence to ethical standards. AI models are tools and should not replace the expertise and judgment of human researchers^[6,7]^.

Likewise, reviewers should disclose to journals if and how AI technology has been used to facilitate their review. Reviewers are reminded that AI can generate possible incorrect, incomplete, or biased material, reinforcing the human factor as still essential for completing the reviewing process^[8]^.

Additionally, ethical concerns apply to AI. Massive amounts of data must be gathered to effectively instruct and use AI, which may come at the cost of patient privacy in most cases. Bias is another concern since AI makes decisions solely on the data it receives as input; this data must represent accurate information.

It is reasonable to expect that the processes involving AI language models for scientific manuscript writing will continue to evolve, being refined, and improved over time, representing an opportunity for scientists to simplify their research process and produce high-quality and impactful articles. The field of AI research is rapidly advancing, and even more advanced language models like ChatGPT are being developed and optimized.

However, the need for skilled researchers and proficient scientific writers is critical to advance science and research ensuring that new discoveries and findings are disseminated effectively. To achieve this goal, it is essential to invest in new researchers’ training and education and afford them the necessary tools and resources to succeed in their endeavors. This includes providing opportunities for research experience using AI as a supporting tool. Therefore, while qualifying researchers and writers for scientific writing using AI can be an innovative and effective approach, it is important to ensure that they have a solid understanding of the principles and conventions of scientific writing. This includes knowledge about types of study design, proper text structuring, peer review, citations, references, and other norms of scholarly style.

The announcement by the ICMJE signals an upheaval in recognizing the potential of AI language models in scientific manuscript writing, provided they are used wisely and in a way that complements human knowledge and skill, considerably advancing scientific understanding.
